# Peripheral T-cell Lymphoma in Japan: Real-World Patient Characteristics, Treatment Patterns, Healthcare Resource Utilization, and Costs

**DOI:** 10.36469/001c.161191

**Published:** 2026-06-11

**Authors:** Charles Dharmani, Oluwatosin Fofah, Pingping Qu, Zoe Jiang, Jingjian Wang, Li Li, Yijing Tao, Lin Song

**Affiliations:** 1 Epidemiology, Clinical Safety and Pharmacovigilance Daiichi Sankyo, Inc.; 2 Data and Statistical Sciences Centre for RWE Daiichi Sankyo, Inc.; 3 Data and Statistical Sciences Centre for Data Engineering Daiichi Sankyo, Inc.; 4 Data and Statistical Sciences Centre for Data Engineering DeltaMed Solutions, Inc; 5 Data Intelligence Department Daiichi Sankyo Co., Ltd

**Keywords:** peripheral T-cell lymphoma, Medical Data Vision, systemic therapy, line of therapy, treatment patterns, healthcare resource utilization, treatment costs

## Abstract

**Background:**

Peripheral T-cell lymphomas (PTCLs) are a rare and heterogeneous group of non-Hodgkin lymphomas associated with aggressive clinical course and suboptimal outcomes. PTCL accounts for a higher proportion of non-Hodgkin lymphoma cases in Japan compared with Western countries; however, real-world evidence on treatment patterns, healthcare resource utilization (HRU), and costs in Japan is limited.

**Objectives:**

To describe real-world patient characteristics, treatment patterns, HRU, and costs among patients with PTCL in Japan.

**Methods:**

This retrospective observational study used administrative claims data from the Medical Data Vision database in Japan. Adult patients diagnosed with PTCL between September 2012 and August 2022 were identified. Patients were categorized as receiving nonsystemic therapy or systemic therapy and stratified by lines of therapy. Patient demographics, comorbidities, treatment regimens, time on treatment, HRU, and healthcare costs, measured per patient per month, were analyzed descriptively.

**Results:**

Among 910 patients identified, 71.3% received systemic treatment and 28.7% received nonsystemic therapy, most receiving corticosteroids only. Mean age at diagnosis was 69.8 years; 70% of patients were at least 65 years old. Key comorbidities included peptic ulcer disease, congestive heart failure, and diabetes. PTCL–not otherwise specified and angioimmunoblastic T-cell lymphoma accounted for over 75% of subtypes. First-line therapy was mainly cyclophosphamide, doxorubicin, vincristine, and prednisone (CHOP) or CHOP-like regimens; later lines were more heterogeneous and of shorter duration. Mogamulizumab and brentuximab vedotin were used in 4.16% and 10.48% of patients, respectively. Stem cell transplantation was infrequent (6.3%). Median per patient per month PTCL-related total healthcare cost was 9712;mediandrug(7056.9) and hospitalization costs ($5177.7) were the main contributors.

**Discussion:**

These findings demonstrate substantial clinical and economic burden among PTCL patients in Japan. Treatment heterogeneity and short duration in later lines of therapy highlight ongoing therapeutic challenges.

**Conclusions:**

High economic burden of PTCL treatment underscores the need for more effective, tolerable, and less costly therapeutic strategies for treating PTCL patients in Japan.

## BACKGROUND

Peripheral T-cell lymphomas (PTCL) are a diverse and clinically aggressive subset of non-Hodgkin lymphomas originating from mature T cells or natural killer cells. PTCLs represent a significant clinical challenge because of their biologic heterogeneity, aggressive clinical course, and suboptimal response to standard therapies.^1^ The most commonly diagnosed subtypes of PTCL include PTCL-not otherwise specified (PTCL-NOS), angioimmunoblastic T-cell lymphoma (AITL), and anaplastic large cell lymphoma (ALCL).^2,3^

In Japan, PTCL comprises approximately 10% to 15% of all non-Hodgkin lymphomas, compared with approximately 5% to 10% in the United States (US) and Europe.^4,5^ The disease is most frequently diagnosed in middle-aged to older adults and demonstrates a male predominance. Patients often present with advanced-stage disease, systemic symptoms, and extranodal involvement, complicating diagnosis and management.^6^ PTCL in Japan is associated with poor prognosis, especially for subtypes like PTCL-NOS and adult T-cell leukemia/lymphoma (ATL).^7,8^

The current standard of care for newly diagnosed PTCL patients remains anthracycline-based chemotherapy regimens such as cyclophosphamide, doxorubicin, vincristine, and prednisone (CHOP). However, the efficacy of CHOP in PTCL is suboptimal, especially in subtypes like PTCL–not otherwise specified (NOS) and AITL.^9,10^ Attempts to improve outcomes with intensive regimens or consolidation with autologous stem cell transplantation have yielded mixed results.^11^ The treatment landscape is further complicated by the lack of targeted therapies and the limited inclusion of PTCL patients in prospective clinical trials.^12,13^

PTCL patients incur a disproportionate economic burden, driven by intensive inpatient care, high drug acquisition costs for both cytotoxic regimens and targeted agents, and frequent use of high-cost procedures such as hematopoietic stem cell transplantation (HSCT) and cellular therapies.^14–17^ Although real-world treatment patterns, HRU, and costs of patients with PTCL in the US have been published^18^; there are no published studies evaluating these in Japan. This study aims to provide a comprehensive overview of PTCL patient demographics, baseline clinical characteristics, real-world treatment patterns, HRU, and costs, overall and by line of therapy (LOT), in Japan using a large, contemporary database.

## METHODS

### Study Design

This real-world observational study retrospectively analyzed a large cohort of patients with PTCL in Japan, utilizing the Medical Data Vision (MDV) database. Due to distinct characteristics and treatment patterns of adult T-cell lymphoma (ATL), a subtype of PTCL, ATL patients were excluded from this cohort. The cohort identification period included data from September 1, 2012, to August 31, 2022, with a 6-month baseline period.

### Data Source

The MDV database is a large, nationwide, hospital-based commercial database containing open claims data for approximately 54.04 million patients across all age groups, as of October 31, 2025. Specifically, MDV includes a considerable proportion of patients over 65 years of age. The database covers approximately 28% of advanced treatment hospitals in Japan, collecting more than 1 million health claims each month.^19^ Furthermore, death information can be obtained from this database, but it is limited to deaths that occur in hospitals contributing data to the MDV database.

Information regarding diseases for hospitalized patients and outpatients includes the relevant *International Classification of Diseases, Tenth Revision* (ICD-10) codes, disease codes, flags indicating the main disease, flags for the disease that consumed the most medical resources, flags for suspicious diagnosis of diseases, and data related to emergencies or accidents. Drug information for hospitalized patients and outpatients encompasses prescription dates, European Pharmaceutical Market Research Association Anatomical Therapeutic Chemical (EPHMRA ATC) classification codes, generic names of drugs, daily doses per health claims, dose units, and the number of days of administration per health claims. Clinical laboratory test values obtained from health claims include the test date, test item codes within MDV based on the Japan Laboratory Analysis Code version 10 (JLAC10), test result values, and test units.

### Patient Population and Index Dates

Adult patients (≥18 years) with at least 1 confirmed claim with earliest diagnosis code (8847430, 8848111, 8840258, 8847274, 8847433, 8847271, 8847416, 8847381, 8847312, 8847400, 8847413, 8847327, 8847417) of PTCL between September 1, 2012, and August 31, 2022, were identified. The date of the first claim of PTCL diagnosis was the index diagnosis date, and a 6-month look-back period prior to the index diagnosis date (baseline period) was determined. Patients were required to be without a non-PTCL primary cancer diagnosis within ±30 days of the index diagnosis date, and to have at least 1 claim during the 6-month look-back period to ensure continuous enrollment. Patients were excluded if there was any claim of systemic PTCL treatment prior to the index diagnosis date. Patients with a diagnosis of ATL were identified and excluded. Disease codes (8847374, 8847375, 8847376, 8847377, 8847378, 8847282, 8835876, 8835877, 8842126) corresponding to ICD-10 codes (C915 and C795) for ATL were used to identify ATL claims in the MDV database. In addition, patients were required to have at least 1 month of continuous follow-up after the index diagnosis date.

### Patient Follow-up and Study Duration

Continuous enrollment (during follow-up) was defined as the presence of at least 1 claim within every 3-month period. The study period for each patient extended from 6 months prior to PTCL diagnosis until death, end of continuous enrollment, or end of follow-up. It should be noted that deaths that occurred outside the hospital and any treatments provided at other healthcare facilities may not have been captured during an individual patient’s follow-up, and the patients were censored at the end of last claim during the study period. Patients were then divided into nonsystemic therapy and systemic therapy cohorts depending on whether patients were given PTCL-related systemic treatments on or after PTCL diagnosis date. The nonsystemic therapy cohort included patients without evidence of systemic therapy for PTCL and included those receiving corticosteroids or supportive care. Systemic treatments were identified using relevant Healthcare Common Procedure Coding System (HCPCS) codes and National Drug Codes (NDC) based on the National Comprehensive Cancer Network guidelines for PTCL treatment.^10^ Patients were considered to have received treatment for PTCL if there was a claim for a drug from these identified systemic therapies.

For the nonsystemic therapy cohort, patients were categorized into steroid and nonsteroid subcohorts, depending on whether patients were given steroid treatment. For the systemic therapy cohort, the date of first PTCL treatment claim was the index treatment date, and patients were required to have at least 1 claim during the 6-month period prior to the index treatment date and to have at least 1 month of continuous follow-up after the index treatment date.

### Line of Therapy

**Figure 1.**
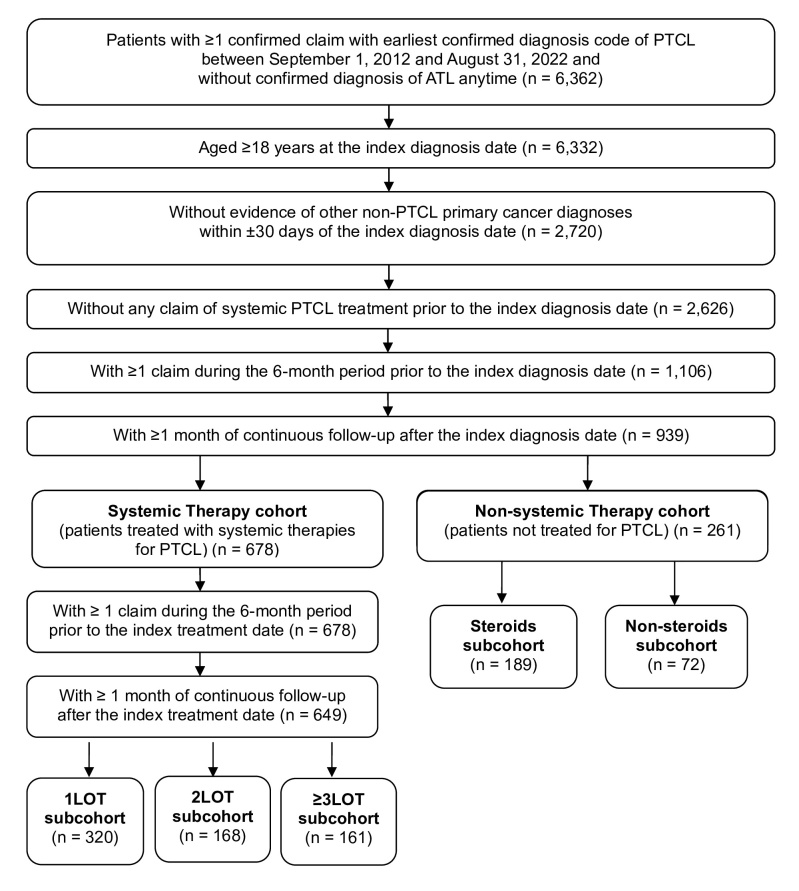
Cohort Selection Criteria and Attrition for Patients with Peripheral T-cell Lymphoma Abbreviations: LOT, line of therapy; PTCL, peripheral T-cell lymphoma.

PTCL-related treatment regimens were identified for the overall systemic therapy cohort and LOT subcohorts during follow-up. Treated patients were subdivided into 3 mutually exclusive subcohorts based on the total number of lines of therapy observed during follow-up: 1LOT, 2LOT, or ≥3LOT **(Figure 1)**. An LOT regimen was defined as a segment of PTCL-related treatment and could be a monotherapy or combination therapy regimen. Among combination regimens, the CHOP-like regimen included combinations with cyclophosphamide, doxorubicin and vincristine while allowing for different corticosteroids (eg, prednisone, methylprednisolone, dexamethasone) within the regimen. Similarly, the CHOEP-like regimen included combinations containing etoposide, cyclophosphamide, doxorubicin and vincristine, along with any corticosteroid. As prednisone is not available in Japan; prednisolone was used for this analysis.

An LOT initiation was defined as the date of the earliest claim for the medication for a monotherapy regimen, or agents for a combination regimen. Any medications given within the 30-day window after the initial medication dispensing belong to the same LOT. An LOT advanced if there was a drug switch or drug augmentation at least 30 days after index treatment initiation. End date for a LOT was defined as the earliest of the following events:

- Discontinuation (gap in treatment of ≥60 days)
- LOT advance (line end date then set to 1 day before line advance)
- End of continuous follow-up
- End of study period or death

### Statistical Analysis

All statistics in this study were descriptive in nature. Continuous variables were summarized using sample size (n), mean, standard deviation (SD), median, 25% percentile (Q1), 75% percentile (Q3), minimum, and maximum. Categorical variables were summarized using frequency and percentage. Missingness in this study primarily reflects structural absence of certain clinical variables in claims data rather than incomplete data capture. Missing data were not imputed. Time on treatment was defined as the duration from LOT initiation to LOT end date based on prespecified rules. Median time on treatment, probability of being on treatment at specific time points and associated 95% confidence intervals (CIs) were estimated using the Kaplan-Meier method stratified by the top treatment regimen observed at first, second, and third LOT.^20,21^ All-cause and PTCL-related HRU and associated costs were assessed in the overall, systemic therapy cohort and LOT subcohorts. HRU and costs were calculated per patient per month (PPPM) from the index treatment date to the end of follow-up. Cost was first calculated in Japanese yen and then converted to US dollars by using the conversion rate on January 1, 2025 (US $1 = ¥157.33).

## RESULTS

### Patient Characteristics

After applying the selection criteria, 910 patients with PTCL were identified. Of these, 649 were included in the systemic therapy cohort and 261 in the nonsystemic therapy cohort. The systemic therapy cohort was further categorized into 1LOT (n = 320), 2LOT (n = 168) and ≥3LOT (n = 161) subcohorts. In the nonsystemic therapy cohort (N = 261), 72% patients were treated with steroids, and 28% had no evidence of receiving any treatment **(Figure 1)**. **Table 1** shows the demographic and clinical characteristics of these PTCL patients.

**Table 1.** Baseline Characteristics of Patients with Peripheral T-cell Lymphoma

**Characteristics**	**Overall (N = 910)**	**Untreated**			**Treated**			
**Overall Untreated (N = 261)**	**Steroids (N = 189)**	**Nonsteroids (N = 72)**	**Overall Treated (N = 649)**	**1LOT (N = 320)**	**2LOT (N = 168)**	**≥3LOT (N = 161)**		
Age at initial diagnosis, years								
Mean (SD)	69.8 (13.8)	72.4 (15.0)	73.0 (14.8)	71.0 (15.4)	68.7 (13.1)	69.5 (13.2)	68.7 (13.4)	67.1 (12.5)
Median	72.0	76.0	77.0	73.5	71.0	72.0	71.0	70.0
Age group at initial diagnosis (years), n (%)								
18-24	4 (0.4)	1 (0.4)	1 (0.5)	0 (0.0)	3 (0.5)	1 (0.3)	2 (1.2)	0 (0.0)
25-39	33 (3.6)	12 (4.6)	8 (4.2)	4 (5.6)	21 (3.2)	7 (2.2)	7 (4.2)	7 (4.3)
40-54	83 (9.1)	20 (7.7)	13 (6.9)	7 (9.7)	63 (9.7)	37 (11.6)	10 (6.0)	16 (9.9)
55-64	129 (14.2)	30 (11.5)	22 (11.6)	8 (11.1)	99 (15.3)	44 (13.8)	28 (16.7)	27 (16.8)
≥65	661 (72.6)	198 (75.9)	145 (76.7)	53 (73.6)	463 (71.3)	231 (72.2)	121 (72.0)	111 (68.9)
Age at index treatment, years								
Mean (SD)	68.7 (13.1)	–	–	–	68.7 (13.1)	69.6 (13.1)	68.7 (13.5)	67.1 (12.5)
Median	71.0	–	–	–	71.0	72.0	71.0	70.0
Sex, n (%)								
Male	524 (57.6)	132 (50.6)	86 (45.5)	46 (63.9)	392 (60.4)	196 (61.3)	98 (58.3)	98 (60.9)
Female	386 (42.4)	129 (49.4)	103 (54.5)	26 (36.1)	257 (39.6)	124 (38.8)	70 (41.7)	63 (39.1)
PTCL subtypes, n (%)								
Peripheral T-cell lymphoma, unspecified	403 (44.3)	125 (47.9)	87 (46.0)	38 (52.8)	278 (42.8)	131 (40.9)	74 (44.0)	73 (45.3)
Angioimmunoblastic T-cell lymphoma	305 (33.5)	62 (23.8)	52 (27.5)	10 (13.9)	243 (37.4)	115 (35.9)	57 (33.9)	71 (44.1)
Extranodal NK/T-cell lymphoma	114 (12.5)	40 (15.3)	25 (13.2)	15 (20.8)	74 (11.4)	48 (15.0)	16 (9.5)	10 (6.2)
Enteropathy-type T-cell lymphoma	19 (2.1)	9 (3.4)	4 (2.1)	5 (6.9)	10 (1.5)	5 (1.6)	4 (2.4)	1 (0.6)
Anaplastic large cell lymphoma	20 (2.2)	3 (1.1)	3 (1.6)	0 (0.0)	17 (2.6)	9 (2.8)	6 (3.6)	2 (1.2)
Subcutaneous panniculitis-like T-cell lymphoma	15 (1.6)	10 (3.8)	8 (4.2)	2 (2.8)	5 (0.8)	2 (0.6)	3 (1.8)	0 (0.0)
Hepatosplenic T-cell lymphoma	10 (1.1)	5 (1.9)	4 (2.1)	1 (1.4)	5 (0.8)	3 (0.9)	1 (0.6)	1 (0.6)
Primary cutaneous anaplastic large cell lymphoma	9 (1.0)	5 (1.9)	4 (2.1)	1 (1.4)	4 (0.6)	2 (0.6)	2 (1.2)	0 (0.0)
≥2 PTCL subtypes	15 (1.6)	2 (0.8)	2 (1.1)	0 (0.0)	13 (2.0)	5 (1.6)	5 (3.0)	3 (1.9)

Abbreviations: LOT, line of therapy; NK, natural killer; PTCL, peripheral T-cell lymphoma; SD, standard deviation.

Across all patients, the mean age at PTCL diagnosis was 69.8 years, with 72.6% being at least 65 years old. The mean age at diagnosis for the systemic therapy cohort was 68.7 years and 72.4 years for the nonsystemic therapy cohort. Patients in the systemic therapy cohort were predominantly male (60.4%), while in the nonsystemic therapy cohort, gender distribution varied in the steroid and nonsteroid groups. The mean (SD) age at index treatment among all treated patients was 68.7 (13.1) years **(Table 1)**.

PTCL-NOS, AITL, and extranodal natural killer/T-cell lymphoma (ENKL) were the most frequently observed subtypes in both the systemic therapy (42.8%, 37.4%, 11.4%, respectively) and nonsystemic therapy cohorts (47.9%, 23.8%, 15.3%, respectively). AITL was more prevalent in the ≥3LOT subcohort compared with 1LOT and 2LOT respectively (44.1% vs 35.9% and 33.9%); ENKL was more prevalent in the 1LOT subcohort compared with 2LOT and ≥3LOT respectively (15.0% vs 9.5% and 6.2%) **(Table 1)**.

A majority of patients had a Charlson Comorbidity Index (CCI) score of 0 (excluding cancer and metastasis) in both the nonsystemic therapy cohort (69.7%) and the systemic therapy cohort (62.9%). Among the nonsystemic therapy and systemic therapy cohorts, the most common comorbidities were peptic ulcer disease (11.1% and 23.1%), congestive heart failure (13.4% and 17.3%), diabetes without chronic complications (6.5% and 15.1%), and mild liver disease (6.9% and 11.4%). Noticeably, the systemic therapy cohort had a higher proportion of each of these comorbidities than the nonsystemic therapy cohort. In the systemic therapy cohort, distributions of a few common comorbidities varied among the three LOT subcohorts. For example, there were seemingly higher proportions of peptic ulcer disease in the 2LOT and ≥3LOT subcohorts (29.2% and 24.2%) than in the 1LOT subcohort (19.4%); the proportion of diabetes without chronic complications varied from 15.3% and 11.9% in the 1LOT and 2LOT subcohorts to 18% in the ≥3LOT subcohort. The prevalence of other CCI comorbidities in the overall cohort was as follows: mild liver disease, 10.1%; renal disease, 6.7%; rheumatologic disease, 4.8%; peripheral vascular disease, 3.4%; dementia, 2.5%; and chronic pulmonary disease, 1.5%.

### Treatment Patterns

In the nonsystemic therapy cohort, 189 PTCL patients (72.4%) received steroid treatment. For the systemic therapy cohort, before initiation of PTCL treatment, prednisolone was the most common steroid treatment (44.7%) followed by dexamethasone and methylprednisolone given to 10.8% and 9.9% of patients, respectively. Within the systemic therapy cohort, 34.2% were given other steroid treatments before initiation of PTCL treatment. In general, a higher proportion of patients received steroids as part of their treatment, as LOT advanced.

The mean time from diagnosis to PTCL treatment initiation for the overall treated cohort was 1.1 months. Median was 0.7 months; however, the maximum was 60.2 months. These findings suggest the presence of potential outliers that may have elevated the mean estimate. Other possible causes may be diagnostic confirmation processes, referral patterns, and clinical workup prior to treatment initiation. The mean time from diagnosis to PTCL treatment initiation was similar for the patients in the 3 LOT subcohorts: 1.1 months, 1.3 months, and 1.1 months for 1LOT, 2LOT, and ≥3LOT, respectively. The mean duration of continuous follow-up for patients included in the overall systemic therapy cohort was 19.8 months (SD: 20.5 months); it was 16.8 months, 18.8 months, and 26.8 months for 1LOT, 2LOT, and ≥3LOT subcohorts, respectively. The median duration of continuous follow-up for the overall cohort was 12.0 months.

Monotherapy regimens were rarely used as the first-line regimen, with etoposide being the most commonly used treatment (1.4%) **(Table 2)**. For combination therapies in the first line, the most common (>5%) regimens included CHOP and CHOP-like (42.8%) therapies, cyclophosphamide + vincristine (20.6%), carboplatin + etoposide + ifosfamide (8.5%), and cyclophosphamide + doxorubicin + etoposide + vincristine (CHOEP and CHOEP-like) (6.2%). These therapies remained the most common first-line regimens for individual LOT subcohorts as well. Mogamulizumab and brentuximab vedotin, as monotherapy or in combination therapies, were used in 0.8% and 5.4% of patients, respectively.

**Table 2.** First-Line Systemic Therapy in Patients with Peripheral T-cell Lymphoma

**Systemic Therapy**	**Overall Treated (N = 649)**	**1LOT (N = 320)**	**2LOT (N = 168)**	**≥3LOT (N = 161)**
1L monotherapy, n (%)^a^				
Etoposide	9 (1.4)	5 (1.6)	2 (1.2)	2 (1.2)
Methotrexate	5 (0.8)	2 (0.6)	1 (0.6)	2 (1.2)
Cyclophosphamide	4 (0.6)	4 (1.3)	0 (0.0)	0 (0.0)
Mogamulizumab	4 (0.6)	0 (0.0)	2 (1.2)	2 (1.2)
Romidepsin	3 (0.5)	3 (0.9)	0 (0.0)	0 (0.0)
1L combination therapy, n (%)^a^				
Cyclophosphamide, doxorubicin, vincristine (CHOP and CHOP-like)	278 (42.8)	136 (42.5)	67 (39.9)	75 (46.6)
Cyclophosphamide, vincristine	134 (20.6)	61 (19.1)	45 (26.8)	28 (17.4)
Carboplatin, etoposide, ifosfamide	55 (8.5)	36 (11.3)	11 (6.5)	8 (5.0)
Cyclophosphamide, doxorubicin, etoposide, vincristine (CHOEP and CHOEP-like)	40 (6.2)	21 (6.6)	5 (3.0)	14 (8.7)
Brentuximab vedotin, cyclophosphamide, doxorubicin	29 (4.5)	19 (5.9)	8 (4.8)	2 (1.2)
Cyclophosphamide, vindesine	16 (2.5)	7 (2.2)	3 (1.8)	6 (3.7)
Etoposide, ifosfamide, methotrexate	12 (1.8)	5 (1.6)	4 (2.4)	3 (1.9)
Cyclophosphamide, cytarabine, etoposide	9 (1.4)	1 (0.3)	3 (1.8)	5 (3.1)
Cyclophosphamide, doxorubicin	5 (0.8)	1 (0.3)	2 (1.2)	2 (1.2)
Cyclophosphamide, etoposide, vincristine	4 (0.6)	2 (0.6)	1 (0.6)	1 (0.6)
Carboplatin, gemcitabine	3 (0.5)	2 (0.6)	0 (0.0)	1 (0.6)
Etoposide, ifosfamide	3 (0.5)	1 (0.3)	2 (1.2)	0 (0.0)

Abbreviations: 1L, first line; LOT, line of therapy.

^a^Regimens with n > 2 in the overall treated cohort.

In the second line, the most commonly utilized (≥2%) monotherapies were etoposide (5.5%), forodesine (4.9%), brentuximab vedotin (4.3%), romidepsin (4.0%) and mogamulizumab (3.6%) **(Table 3)**. Slightly more patients used combination therapy such as carboplatin + etoposide + ifosfamide (7.9%), CHOEP and CHOEP-like therapies (6.1%), carboplatin + gemcitabine (4.6%), cisplatin + gemcitabine (4.6%), and cyclophosphamide + cytarabine + etoposide (4.3%). Mogamulizumab and brentuximab vedotin, as monotherapy or in combination therapies, were used in 4.9% and 8.5% of patients, respectively.

**Table 3.** Second-Line Systemic Therapy in Patients with Peripheral T-cell Lymphoma

**Systemic Therapy**	**Overall Treated ≥2LOT (N=329)**	**1LOT (N=320)**	**2LOT (N=168)**	**≥3LOT (N=161)**
2L monotherapy, n (%)^a^				
Etoposide	18 (5.5)	–	10 (6.0)	8 (5.0)
Forodesine	16 (4.9)	–	8 (4.8)	8 (5.0)
Brentuximab vedotin	14 (4.3)	–	7 (4.2)	7 (4.3)
Romidepsin	13 (4.0)	–	8 (4.8)	5 (3.1)
Mogamulizumab	12 (3.6)	–	7 (4.2)	5 (3.1)
Methotrexate	6 (1.8)	–	4 (2.4)	2 (1.2)
Pralatrexate	6 (1.8)	–	3 (1.8)	3 (1.9)
Cisplatin	3 (0.9)	–	3 (1.8)	0 (0.0)
2L combination therapy, n (%)^a^				
Carboplatin, etoposide, ifosfamide	26 (7.9)	–	14 (8.3)	12 (7.5)
Cyclophosphamide, doxorubicin, etoposide, vincristine (CHOEP and CHOEP-like)	20 (6.1)	–	10 (6.0)	10 (6.2)
Carboplatin, gemcitabine	15 (4.6)	–	4 (2.4)	11 (6.8)
Cisplatin, gemcitabine	15 (4.6)	–	5 (3.0)	10 (6.2)
Cyclophosphamide, cytarabine, etoposide	14 (4.3)	–	6 (3.6)	8 (5.0)
Cyclophosphamide, vincristine	12 (3.6)	–	7 (4.2)	5 (3.1)
Cyclophosphamide, doxorubicin, vincristine (CHOP and CHOP-like)	11 (3.3)	–	7 (4.2)	4 (2.5)
Cisplatin, cytarabine, etoposide	8 (2.4)	–	2 (1.2)	6 (3.7)
Cyclophosphamide, vindesine	8 (2.4)	–	4 (2.4)	4 (2.5)
Cytarabine, methotrexate	8 (2.4)	–	6 (3.6)	2 (1.2)
Cytarabine, etoposide, ranimustine	7 (2.1)	–	5 (3.0)	2 (1.2)
Brentuximab vedotin, cyclophosphamide, doxorubicin	6 (1.8)	–	4 (2.4)	2 (1.2)
Carboplatin, cyclophosphamide, etoposide, ranimustine	5 (1.5)	–	2 (1.2)	3 (1.9)
Etoposide, ifosfamide, methotrexate	5 (1.5)	–	1 (0.6)	4 (2.5)
Cyclophosphamide, etoposide, vincristine	4 (1.2)	–	0	4 (2.5)
Carboplatin, cytarabine, etoposide	3 (0.9)	–	1 (0.6)	2 (1.2)
Cyclophosphamide, etoposide	3 (0.9)	–	2 (1.2)	1 (0.6)
Etoposide, ifosfamide	3 (0.9)	–	3 (1.8)	0
Etoposide, vindesine	3 (0.9)	–	1 (0.6)	2 (1.2)

Abbreviations: 2L, second line; LOT, line of therapy.

^a^Regimens with n>2 in the overall treated ≥2 LOT cohort.

In the third line, the most common (≥2%) monotherapies were romidepsin (9.9%), forodesine (7.5%), pralatrexate (4.3%), etoposide (3.1%), and cyclophosphamide (2.5%). The most common (>3%) combination therapies were cisplatin + gemcitabine (7.5%), carboplatin + etoposide + ifosfamide (5.6%), carboplatin + gemcitabine (4.3%), cyclophosphamide + cytarabine + etoposide (3.1%), CHOEP and CHOEP-like (3.1%), cyclophosphamide + vincristine (3.1%) **(Table 4)**. Mogamulizumab and brentuximab vedotin, as monotherapy or in combination therapies, were used in 3.7% and 3.1% of patients, respectively. Mogamulizumab and brentuximab vedotin were administered as monotherapy or in combination therapy in 4.16% and 10.48% of patients across all treatment lines.

**Table 4.** Third-Line Systemic Therapy in Patients with Peripheral T-Cell Lymphoma

**Systemic Therapy**	**Overall Treated (N=649)**	**1LOT (N=320)**	**2LOT (N=168)**	**≥3LOT (N=161)**
3L monotherapy, n (%)^a^				
Romidepsin	–	–	–	16 (9.9)
Forodesine	–	–	–	12 (7.5)
Pralatrexate	–	–	–	7 (4.3)
Etoposide	–	–	–	5 (3.1)
Cyclophosphamide	–	–	–	4 (2.5)
Cytarabine	–	–	–	3 (1.9)
Methotrexate	–	–	–	3 (1.9)
Mogamulizumab	–	–	–	3 (1.9)
3L combination therapy, n (%)^a^				
Cisplatin, gemcitabine	–	–	–	12 (7.5)
Carboplatin, etoposide, ifosfamide	–	–	–	9 (5.6)
Carboplatin, gemcitabine	–	–	–	7 (4.3)
Cyclophosphamide, cytarabine, etoposide	–	–	–	5 (3.1)
Cyclophosphamide, doxorubicin, etoposide, vincristine (CHOEP and CHOEP-like)	–	–	–	5 (3.1)
Cyclophosphamide, vincristine	–	–	–	5 (3.1)
Cyclophosphamide, doxorubicin, vincristine (CHOP and CHOP-like)	–	–	–	4 (2.5)
Cyclophosphamide, etoposide	–	–	–	4 (2.5)
Cytarabine, etoposide, ranimustine	–	–	–	4 (2.5)
Cisplatin, cytarabine, etoposide	–	–	–	3 (1.9)
Cytarabine, methotrexate	–	–	–	3 (1.9)

Abbreviations: 3L, third line; LOT, line of therapy.

^a^Regimens with n >2 in the ≥3LOT cohort.

Forty-one (6.3%) patients in the treated cohort underwent stem cell transplantation, with the highest proportion observed among ≥3LOT subgroup patients (16.2%), followed by 2LOT (7.7%) and 1LOT subcohorts (0.6%). Overall, autologous HSCT was more common (5.2%), followed by cord blood (0.9%) and allogeneic HSCT (0.2%). Among autologous HSCTs, 35% were done in patients in 2LOT and 64.7% in those in ≥3LOT subgroup.

For the top 5 treatment regimens in the first LOT, the median time on treatment was 3.5 months (95% CI, 3.5-3.6 months) for cyclophosphamide + doxorubicin + vincristine, 2.6 months (95% CI, 1.9-3.4 months) for cyclophosphamide + vincristine, 1.5 months (95% CI, 1.5-1.7 months) for carboplatin + etoposide + ifosfamide, 3.6 months (95% CI, 2.8-4.3 months) for brentuximab vedotin + cyclophosphamide + doxorubicin, and 2.4 months (95% CI, 1.7-3.8 months) for cyclophosphamide + doxorubicin + etoposide + vincristine. At 3 months, the probabilities of patients still being on treatment were 62.0%, 44.8%, 3.6%, 69.0%, and 44.8% for the top 5 treatment regimens respectively. At 6 months, their probabilities dropped to 2.7%, 1.5%, 0%, 6.9%, and 0%, respectively. In the second LOT, the most frequently observed regimen was carboplatin + etoposide + ifosfamide, for which the median time on treatment was 1.5 months (95% CI, 0.8-2.4 months). In the third LOT, the most frequently observed regimen was romidepsin, for which the median time on treatment was 2.0 months (95% CI, 0.0-3.9 months).

Of all patients in the systemic therapy cohort, nearly 92% discontinued their first-line treatment, 43.5% discontinued their second-line treatment, and 21.7% discontinued their third-line treatment. Also, of all patients in the systemic therapy cohort, nearly 8% switched their first-line treatment, 7.2% switched their second-line treatment, and 3.1% switched their third-line treatment.

### Healthcare Resource Utilization and Costs

During the baseline period, mean all-cause healthcare costs in PTCL patients were **¥**565 000 ($3591) PPPM, with a median of **¥**176 700 ($1123). Costs were higher for patients receiving advanced lines of therapy, with mean values of **¥**527 700 ($3354), **¥**593 700 ($3774), and **¥**609 300 ($3873) PPPM for patients in 1LOT, 2LOT, and ≥3LOT, respectively. The corresponding medians were **¥**149 600 ($951), **¥**218 800 ($1391), and **¥**178 200 ($1133) PPPM.

Over 98% of patients in the systemic therapy cohort had PTCL-related hospitalizations, with the mean number of hospitalizations per month being 0.4. Similar estimates were observed for the three LOT subcohorts for hospitalizations (97.8%, 100%, 98.1%) and mean numbers of hospitalizations per month (0.4, 0.5, 0.4). Among patients who were hospitalized, the mean length of stay per hospitalization was 1.3 months with similar length of stay for the 3 LOT subcohorts (1.3, 1.3, and 1.2 months, respectively).

Overall, 33.4% patients of the systemic therapy cohort had at least 1 PTCL-related emergency room (ER) visit (1LOT, 23.8%; 2LOT, 44%; ≥3LOT, 41.6%). Overall, the mean number of ER visits per month was 0.2, or 2 over 10 months, with the PPPM mean decreasing in patients on higher lines of therapy (1LOT, 0.3; 2LOT, 0.2; 3LOT, 0.1). PTCL patients included in the advanced LOT cohorts presumably have tolerated their initial lines of therapies; hence, it is likely that there were fewer complication-related ER visits during the course of their earlier lines. In their later lines, patients may have higher complication-related ER visits. However, as ER visits PPPM from the date of initial treatment are being averaged, their mean ER visit estimates are likely to be lower.

Nearly 80% of the systemic therapy cohort had PTCL-related outpatient visits, which increased in patients on higher lines of therapy, from 72.2% in 1LOT to 90.1% in 3LOT. Among patients who had PTCL-related outpatient visits, the mean number of outpatient visits was 1.5 per month with similar estimates in the 3 subcohorts (1.4, 1.6, 1.5 times per month). The mean number of PTCL-related prescription drugs per month was 0.8 (SD, 0.9), with a decreasing PPPM mean with increasing LOT in the 3 subcohorts (1.0, 0.8, 0.6). The distributions of all-cause HRU were similar to those of the PTCL-related HRU suggesting that the patients used HRU mostly for PTCL-related reasons.

For the systemic therapy cohort, median all-cause healthcare costs during the baseline period were ¥176,700 ($1123) overall, and ¥149 600 ($951), ¥218 800 ($1391), and ¥178 200 ($1133) for the 1LOT, 2LOT, and ≥3LOT subcohorts, respectively. The median PTCL-related total healthcare cost PPPM was ¥1 528 000 ($9712). These median costs increased with advancing LOT, from ¥1 163 500 ($7395) for 1LOT to ¥2 188 800 ($13 912) for 2LOT and ¥2 078 100 ($13 208) for ≥3LOT.

PTCL-related costs of hospitalizations, ER visits, outpatient visits, prescription drugs, stem cell transplants, all drugs (prescription and nonprescription), and labs, overall and by LOT PPPM are described in **Table 5**. Cumulative mean cost of total PTCL-related healthcare PPPM was ¥3 191 000 ($20 282). It varied from ¥2 012 500 ($12 791) for 1LOT to ¥4 870 300 ($30 956) for ≥3LOT patients. Cumulative mean cost of total PTCL-related healthcare per patient was ¥57 688 400 ($366 635). It varied from ¥23 684 600 ($150 528) for 1LOT to ¥122 877 900 ($780 991) for ≥3LOT patients (**Table 5**).

**Table 5.** PTCL-Related Treatment Costs in Patients with Peripheral T-cell Lymphoma

**Treatment Costs**	**Overall Treated (N = 649)**	**1LOT (N = 320)**	**2LOT (N = 168)**	**≥3LOT (N = 161)**
Cost of hospitalizations PPPM (in ¥1000)				
n (%)	639 (98.5)	313 (97.8)	168 (100)	158 (98.1)
Mean (SD)	¥1620.0 (3260.8) $10 296.8 (20 725.9)	¥1231.5 (1845.7) $7827.5 (11 731.4)	¥1885.5 (2871.9) $11 984.4 (18 254.0)	¥2107.4 (5201.1) $13 394.8 (33 058.5)
[Q1, Q3]	¥[215.0, 1854.3] $[1366.6, 11 786.1]	¥[125.3, 1542.0] $[796.4, 9801.1]	¥[327.2, 2279.6] $[2079.7, 14 489.3]	¥[452.9, 2013.4] $[2878.7, 12 797.3]
Cost of ER visits (¥1000 PPPM)				
n (%)	214 (33.0)	75 (23.4)	74 (44.0)	65 (40.4)
Mean (SD)	¥736.6 (1159.0) $4682.8 (7364.1)	¥951.6 (1457.7) $6047.9 (9265.4)	¥688.5 (937.5) $4374.1 (5958.8)	¥543.1 (957.0) $3451.8 (6083.3)
[Q1, Q3]	¥[49.8, 871.8] $[316.5, 5541.2]	¥[47.3, 1036.2] $[300.6, 6586.2]	¥[42.6, 968.5] $[270.8, 6155.9]	¥[67.7, 422.9] $[430.3, 2688.0]
Cost of outpatient visits (¥1000 PPPM)				
n (%)	516 (79.5)	231 (72.2)	140 (83.3)	145 (90.1)
Mean (SD)	¥1982.5 (4885.5) $12 600.9 (31 052.6)	¥1110.5 (2388.7) $7058.4 (15 182.7)	¥2356.6 (5016.3) $14 978.7 (31 883.9)	¥3010.6 (7038.4) $19 135.6 (44 736.5)
[Q1, Q3]	¥[145.2, 1507.2] $[922.9, 9579.9]	¥[118.3, 906.5] $[751.9, 5761.8]	¥[193.0, 2488.9] $[1226.7, 15 819.6]	¥[201.8, 1809.4] $[1282.7, 11 500.7]
Cost of prescription drugs^a^ (¥1000 PPPM)				
n (%)	645 (99.4)	319 (99.7)	168 (100)	158 (98.1)
Mean (SD)	¥835.1 (3765.5) $5308.0 (23 933.8)	¥313.8 (1206.5) $1994.5 (7668.6)	¥831.7 (3399.4) $5286.3 (21 606.8)	¥1891.2 (6422.4) $12 020.6 (40 821.2)
[Q1, Q3]	¥[24.6, 286.1] $[156.4, 1818.5]	¥[12.2, 96.0] $[77.5, 610.2]	¥[36.1, 472.3] $[229.5, 3002.0]	¥[76.4, 762.6] $[485.6, 4847.1]
Cost of stem cell transplants (¥1000 PPPM)				
n (%)	41 (6.3)	2 (0.6)	14 (8.3)	25 (15.5)
Mean (SD)	¥27.5 (29.0) $174.8 (184.3)	¥3.8 (5.3) $24.2 (33.7)	¥32.7 (32.4) $207.8 (205.9)	¥26.4 (27.7) $167.8 (176.1)
[Q1, Q3]	¥[7.8, 41.0] $[49.6, 260.6]	¥[0.06, 7.6] $[0.38, 48.3]	¥[7.8, 46.5] $[49.6, 295.6]	¥[10.8, 29.3] $[68.6, 186.2]
Cost of drugs^b^ (¥1000 PPPM)				
n (%)	645 (99.4)	319 (99.7)	168 (100)	158 (98.1)
Mean (SD)	¥2776.0 (5514.0) $17 644.4 (35 047.4)	¥1611.9 (2543.4) $10 245.3 (16 166.0)	¥3389.3 (5388.7) $21 542.6 (34 250.9)	¥4474.3 (8641.1) $28 438.9 (54 923.4)
[Q1, Q3]	¥[457.1, 2682.1] $[2905.4, 17 047.6]	¥[290.4, 1721.6] $[1845.8, 10 942.6]	¥[744.7, 4048.3] $[4733.4, 25 731.3]	¥[757.2, 3480.5] $[4812.8, 22 122.3]
Cost of labs (¥1000 PPPM)				
n (%)	643 (99.1)	317 (99.1)	168 (100)	158 (98.1)
Mean (SD)	¥47.4 (52.5) $301.3 (333.7)	¥44.5 (58.4) $282.8 (371.2)	¥53.7 (54.9) $341.3 (348.9)	¥46.3 (34.0) $294.3 (216.1)
[Q1, Q3]	¥[15.5, 61.8] $[98.5, 392.8]	¥[11.4, 55.5] $[72.5, 352.8]	¥[18.4, 69.6] $[117.0, 442.4]	¥[22.8, 59.5] $[144.9, 378.2]
Cost of total^c^ healthcare PPPM (¥1000 PPPM)				
n (%)	645 (99.4)	319 (99.7)	168 (100)	158 (98.1)
Mean (SD)	¥3191.0 (5562.3) $20 282.2 (35 354.4)	¥2012.5 (2667.7) $12 791.6 (16 956.1)	¥3849.4 (5450.7) $24 467.0 (34 645.0)	¥4870.3 (8647.1) $30 956.0 (54 961.5)
[Q1, Q3]	¥[786.9, 3061.6] $[5001.6, 19 459.7]	¥[473.4, 2527.8] $[3009.0, 16 066.9]	¥[1135.8, 4380.9] $[7219.2, 27 845.3]	¥[1077.9, 4190.8] $[6851.2, 26 637.0]
Cumulative^d^ cost of PTCL-related healthcare per patient (¥1000)				
n (%)	645 (99.4%)	319 (99.7%)	168 (100%)	158 (98.1%)
Mean (SD)	¥57 688.4 (163 546.8) $366 635.3 (1 039 446.6)	¥236 84.6 (65 599.0) $150 528.3 (416 956.0)	¥60 938.8 (148 392.8) $387 289.2 (943 296.3)	¥122 877.9 (266 281.9) $780 991.3 (1 692 677.1)
[Q1, Q3]	¥[6126.4, 40 494.4] $[38 944.2, 257 435.4]	¥[3776.2, 17 957.4] $[23 999.9, 114 139.7]	¥[9966.2, 51 576.8] $[63 333.8, 327 873.6]	¥[17 913.0, 81 749.2] $[113 874.9, 519 645.9]

Abbreviations: ER, emergency room; LOT, line of therapy; PPPM, per patient per month; Q, quartile; SD, standard deviation.

^a^ Prescription drugs include drugs for PTCL-related anticancer treatments (excluding steroids).

^b^ Drugs include all drugs including anticancer drugs.

^c^ Total healthcare includes all healthcare utilization including hospitalizations, ER visits, outpatient visits, prescription drugs, stem cell transplants, prescription and non-prescription drugs, and labs.

^d^ From diagnosis to end of follow-up.

The total PTCL-related healthcare cost was analyzed in two ways for further assessment: (1) by drug cost, lab cost, and other cost, including cost for stem cell transplant, and (2) by inpatient cost and outpatient cost, where ER cost was part of the inpatient cost. In the first approach, drugs (both PTCL and non-PTCL-related) were a major cost driver with a median of ¥1 110 278 ($7057) PPPM for the overall treated cohort, accounting for 73% ([$7056.9 / $9711.7] * 100%) of the total cost PPPM, with increasing cost with increasing LOT, and being highest for the ≥3LOT subcohort (¥1 552 060; $9865). The median cost of prescription drugs was ¥72 844 ($463) PPPM. In the second approach, the median cost for hospitalizations (inpatient visits) and outpatient visits were ¥814 655 ($5178) PPPM and ¥505 344 ($3212) PPPM, respectively, with generally increasing cost with increasing LOT. The PTCL-related healthcare cost was 93% ([$9711.7/$10 414.8] * 100%) of all-cause healthcare cost. The cost patterns for all-cause HRU were similar to those of the PTCL-related healthcare cost.

From PTCL diagnosis until end of follow-up, all-cause costs of hospitalizations, ER visits, outpatient visits, prescription drugs, stem cell transplants, all drugs (prescription and non-prescription), and labs, overall and by LOT, per patient are described in **Supplementary Table S1**. All-cause mean cost of healthcare PPPM was ¥3 341 400 ($21 238). It varied from ¥2 214 500 ($14 075) for 1LOT to ¥4 936 000 ($31 373) for ≥3LOT patients. The overall, all-cause cumulative mean cost of healthcare per patient was ¥62 323 900 ($396 149). Mean costs were lowest among patients in 1LOT ($179 685 [¥28 269 841]), which more than doubled for 2LOT ($416 065 [¥65 459 506]), and were highest among patients receiving ≥3LOT ($805 607 [¥126 746 149]). Median costs demonstrated a similar increase, rising from $47 655 (¥7 496 361) for 1LOT to $131 207 (¥20 643 598) and $263 921 (¥41 519 295) for 2LOT and ≥3LOT, respectively.

## DISCUSSION

This is the first study to leverage one of the largest healthcare databases in Japan to evaluate patient characteristics, treatment patterns, HRU, and costs among PTCL patients in Japan. In this study, the cohort was predominantly male (57.6%), with a mean age of 69.8 years, aligning with previous publications.^4^ Among patients who received no systemic therapy but only received steroids, 54.5% were female. This observed difference in sex distribution may reflect differences in treatment selection or underlying patient characteristics; however, these factors cannot be assessed in this analysis. Nearly, 65% of all PTCL patients had a CCI score of 0 at baseline. Considering that the mean age of this study population is approximately 70 years, this proportion is high for the Japanese population of this age group. This suggests that patient comorbidities may not be fully captured in the MDV database; hence, these estimates should be interpreted with caution.

In this study, PTCL-NOS (44.3%), AITL (33.5%) and ENKL (12.5%) were the most frequently observed subtypes, whereas ALCL was only observed in 2.2% of patients. Similar distribution has been reported in Japan and other Asian countries.^22^ This differs from that reported in the US where ALCL and PTCL-NOS were the two most common PTCL subtypes (40.9% and 48.0%, respectively).^18^ Subtype-specific diagnoses may be undercoded or misclassified in claims data, with some cases potentially coded as PTCL NOS. Additionally, the hospital-based nature of the MDV database may influence the observed distribution of subtypes.

In this real-world analysis, the majority of the PTCL patients (71.3%) received systemic treatment; many (20.8%) received only supportive care in the form of corticosteroids, and a substantial minority (7.9%) received no treatment of any kind. Compared with the US,^18^ a significantly higher proportion of PTCL patients in Japan are treated with systemic therapy and steroids. However, this comparison should be interpreted with caution due to differences in healthcare systems, database structure, and coding practices. In particular, the MDV database, which is hospital-based, may overrepresent inpatient care and influence estimates of hospitalization, treatment patterns, and HRU.

The first-line treatment was dominated by CHOP or CHOP-like regimens (42.8%), followed by cyclophosphamide + vincristine combinations (20.6%) and carboplatin–etoposide–ifosfamide regimens (8.5%). These findings broadly align with estimates reported from the US.^18^ Monotherapy regimens were rarely used in the first line (5.1%), most commonly etoposide (1.4%). However, a US real-world study reported that 37.03% patients were treated with various monotherapy regimens in the first line.^18^ Similarly, in the second line, monotherapy was used in 29.2% of patients compared with 39% as reported by the US real-world study.^18^ In the third line, monotherapy was used in only 37.3% patients; corresponding estimates of 74.2% have been reported from the US.^18^ Reasons for this substantial difference in the use of monotherapies in all lines of therapy among PTCL patients in the Japan vs US needs further evaluation.

In this study, the newer treatments, mogamulizumab and brentuximab vedotin were used as monotherapy or in combination regimens in 4.16% and 10.48% of patients across all lines of therapy, respectively. Comparatively, across all lines of therapy, brentuximab was used as monotherapy in 13.8% to 19.4% patients and in only 5.8% of patients in combination regimens in the US real-world study.^18^ These newer therapies were approved in Japan after 2014; the present study included data until 2022. This observed difference in use of biologic and targeted therapies should be interpreted with caution and potentially could be due to differences in drug approval dates, patient characteristics and treatment practice patterns.

Prior to initiation of systemic therapy, almost all (99.5%) patients were treated with steroids compared with only 67% in the US real-world study.^18^ Similarly, 20.8% of the overall cohort received steroids only, underscoring a potentially palliative intent in a substantial subset of patients. Further research needs to be conducted to understand the reasons why such a substantial proportion (28.7%) of PTCL patients are not given any treatment or are just given steroids in Japan.

Stem cell transplantation was infrequent (6.3%), with autologous transplantation being the predominant modality. Stem cell transplant utilization was the most common in the ≥3LOT (16.2%) subcohort. A recent US study of patients with PTCL found a higher utilization of stem cell transplantation (17%) with a high proportion observed among ≥3LOT patients (22.6%).^18^

This observed difference is likely due to a difference in the age distribution of PTCL patients in our study (mean 69.8 years) compared with PTCL patients in the US study (mean, 53.5 years). This difference can be explained by the fact that US commercial claims data cover a younger patient population than the MDV database. Also, it is possible that HSCT is not sufficiently captured in the MDV database or the proportion of patients undergoing HSCT is lower in Japan than in the US; the reasons for this should be evaluated in future research.

Discontinuation rates in the 1LOT, 2LOT and ≥3LOT subcohorts were 100%, 78.6%, and 90.1%, respectively. These results differ from a recent US study that reported 67.5%, 53.7%, and 51.6% of patients discontinuing in 1LOT, 2LOT, and ≥3LOT subgroups, respectively.^18^ Observed discontinuation may be due to a combination of factors, including disease progression, treatment-related toxicity, death, loss to follow-up, or censoring at the end of follow-up, which cannot be distinguished in this study.

In this study, PTCL patients had an estimated average of 0.4 hospitalizations per month. Although this estimate is high, it is likely that the patients included in this study cohort are at a more advanced stage of the disease, given that MDV database predominantly contains data on inpatient care rather than outpatient visits. In addition, a large proportion of cancer patients in Japan receive systemic therapy in the hospital setting rather than outpatient setting.

The mean all-cause healthcare cost for the systemic therapy cohort was $3591.2 PPPM during the baseline period. This high baseline cost highlights the substantial healthcare needs of PTCL patients in Japan even prior to the initiation of systemic therapy. The median PTCL-related total healthcare cost was $9711.70 PPPM (mean, $20 282.20) and increased with advancing lines of therapy; it was substantially higher than those reported from the US (mean, $17 251).^18^ Cost data in oncology care are typically right-skewed, with a subset of patients incurring disproportionately high costs. This is reflected in the large standard deviations observed for cost estimates observed in this study and highlights the importance of considering both mean and median values when interpreting these estimates.

Several observations emerge from these findings. First, drug costs were the predominant cost driver, accounting for approximately 73% of PTCL-related expenses (median, $7056.90 PPPM). Costs attributable to non-PTCL-specific drugs were low (median, $463 PPPM); however, these costs may be underestimated due to incomplete capture of outpatient supportive care and medications obtained outside participating hospitals. Second, the high inpatient costs (median, $5177.70 PPPM) relative to outpatient costs (median US $3212.30 PPPM) highlight the burden of hospitalization (including ER admissions) as a major contributor to total cost. Importantly, 93% of the all-cause healthcare cost was PTCL-related healthcare cost, indicating that nearly the entire expense burden in these patients was attributable to the treatment of the disease.

These findings highlight the substantial clinical and economic burden associated with PTCL in Japan. However, these results should be interpreted in the context of a hospital-based claims database, which may not fully reflect national treatment patterns. Further research is needed to evaluate how different treatment strategies may impact outcomes and healthcare costs. As drug and inpatient costs are the primary cost drivers in this patient population, improved utilization of interventions that are administered in the outpatient setting, minimizing hospital stays, and improved supportive care may reduce the economic burden. As newer targeted agents, such as valemetostat, and immunotherapies are developed for PTCL treatment and adopted, real-world evaluations should not only assess their efficacy and safety but also evaluate means to offset their treatment costs.^23^

Although this study utilized the largest Japanese claims database, several inherent limitations arise from the use of administrative claims data. Key clinical variables, such as disease stage, tumor burden, performance status, and treatment response, are not captured, limiting the ability to assess certain outcomes, including disease progression, and to fully contextualize treatment selection and healthcare utilization; these factors may influence treatment choice and associated outcomes.

The hospital-based nature of the MDV database, which primarily includes large acute-care institutions, may overrepresent patients with more severe disease and inpatient care, limiting generalizability to broader real-world clinical practice in Japan. Longitudinal follow-up is also restricted to participating hospitals, and care received across multiple institutions may not be fully captured, potentially resulting in incomplete ascertainment of subsequent lines of therapy, supportive care, and overall HRU.

As this was a descriptive study, adjusted analyses were not conducted to account for confounding factors; this may influence the estimates of the variables being assessed. As there are several subtypes of PTCL, and many of them are rare, it is challenging to conduct stratified analysis by subtype due to sample size restrictions. This study presents evaluation of PTCL patient cohort, including all subtypes; however, patient characteristics, treatment patterns, HRU and costs are likely to vary by subtype.

The requirement of at least 1 claim during the 6-month baseline period does not fully ensure continuous observation. Unlike most closed claims databases in the US, MDV database does not include an enrollment variable. Consequently, it is challenging to capture patients’ continuous medical records given the characteristics of the MDV database. Limiting regimen combination grouping to a 30-day period may lead to some regimens that are intended for combination therapy, but may be given asynchronously, being misclassified. Nevertheless, most standard combination therapies used in PTCL treatment are administered within the 30-day timeframe, thereby minimizing the risk of such misclassification. As the MDV database is inpatient-oriented, it is likely that estimates of hospitalization, steroid use, and combination therapy vs monotherapy were overestimated in this study cohort; the extent of this overestimation cannot be ascertained in the current analysis.

As with all claims-based analyses, measurement of treatment exposure and clinical outcomes, such as treatment response or progression, is subject to data capture limitations. In this study, patients were censored at their last observed claim, which may be considered as a loss to follow-up or true treatment discontinuation, potentially leading to underestimation of time on treatment; the extent of this underestimation cannot be ascertained. Also, reasons for treatment discontinuation (eg, disease progression, toxicity, or death) cannot be determined. Additionally, HRU and costs may be underestimated, as services and prescriptions outside participating hospitals are not fully captured. Coding practices and errors in administrative claims data may introduce misclassification or bias, as diagnoses and treatments are recorded for billing rather than research purposes, and may be inconsistently or incompletely captured. Finally, the comparison of the results of this study with the results from the US claims data study^18^ is for contextualization. The differences in the results of these studies may be due to variability in several factors, including patient population included and their characteristics, databases used and their type and coverage, coding practices, extent of data missingness, and country-specific clinical practice and reimbursement policies.

## CONCLUSIONS

Real-world treatment patterns of PTCL patients in Japan largely reflect recommended treatment guidelines; however, nearly one-third of patients do not receive systemic therapy and instead receive corticosteroids alone for supportive care. CHOP-based regimens remain the predominant frontline approach, while treatment beyond the first line is heterogeneous, short in duration, and associated with low rates of stem cell transplantation. PTCL patients incur substantial healthcare resource utilization and treatment costs in Japan. Further research is warranted to better understand treatment outcomes, economic burden, and the impact of evolving treatment approaches among PTCL patients in the real-world setting.

### Disclosures

C.D., O.F., P.Q., and Z.J. are employees of Daiichi Sankyo, Inc. J.W., L.L., and Y.T. are employees of DeltaMed Solutions, Inc. L.S. is an employee of Daiichi Sankyo Company, Limited, Japan. C.D., O.F., P.Q, and Z.J. own restricted stock units of Daiichi Sankyo, Inc.

## Supplementary Material

Online Supplementary Material
