# Supplementary material for: Peripheral T-cell Lymphoma in Japan: Real-World Patient Characteristics, Treatment Patterns, Healthcare Resource Utilization, and Costs
Source: J Health Econ Outcomes Res. 2026 Jun 11;13(1):161191. doi: 10.36469/001c.161191 (PMC13264034; doi:10.36469/001c.161191)
Supplement: Online Supplementary Material [file jheor_2026_13_1_161191_341968.pdf]

Journal of Health Economics and Outcomes Research

Online Supplementary Material

Peripheral T-cell Lymphoma in Japan: Real-World Patient Characteristics, Treatment Patterns, Healthcare Resource Utilization, and Costs. *JHEOR*. 2026;13(1):??-??. doi:10.36469/jheor.2026.161191

Table S1: Overall All-Cause Treatment Costs in Patients with Peripheral T-cell Lymphoma

This supplementary material has been provided by the authors to give readers additional information about their work.

This is an open-access article distributed under the terms of the Creative Commons Attribution 4.0 International License (CCBY-4.0). View this license's legal deed at http://creativecommons.org/licenses/by/4.0 and legal code at http://creativecommons.org/licenses/by/4.0/legalcode for more information.

Table S1. Overall All-Cause Treatment Costs in Patients with Peripheral T-cell Lymphoma

**Treatment Costs**	**Overall Treated (N = 649)**	**1LOT (N = 320)**	**2LOT (N = 168)**	**≥3LOT (N = 161)**
Cost of hospitalizations PPPM (in ¥1000)				
n (%)	643 (99.1)	314 (98.1)	168 (100)	161 (100.0)
Mean (SD)	¥1687.4 (3370.5)<br>$10 725.2 (21423.1)	¥1333.4 (2244.3)<br>$8475.2 (14 264.9)	¥1948.2 (2889.0)<br>$12 382.9 (18 362.7)	¥2105.7 (5150.2)<br>$13 384.0 (32 735.0)
[Q1, Q3]	¥[225.7, 1902.6]<br>$[1434.6, 12 093.1]	¥[130.0, 1603.9]<br>$[826.3, 10 194.5]	¥[332.9, 2331.3]<br>$[2115.9, 14 817.9]	¥[488.5, 2013.4]<br>$[3104.9, 12 797.3]
Cost of ER visits (¥1000 PPPM)				
n (%)	217 (33.4)	76 (23.8)	74 (44.0)	67 (41.6)
Mean (SD)	¥730.9 (1153.3)<br>$4646.6 (7327.1)	¥947.4 (1451.1)<br>$6021.2 (9222.7)	¥689 (937.2)<br>$4377.2 (5956.9)	¥531.7 (944.9)<br>$3379.4 (6004.3)
[Q1, Q3]	¥[55.2, 871.8]<br>$[350.9, 5541.2]	¥[48.5, 1068.7]<br>$[308.3, 6792.7]	¥[42.6, 968.5]<br>$[270.8, 6155.9]	¥[64.8, 422.9]<br>$[411.9, 2688.0]
Cost of outpatient visits (¥1000 PPPM)				
n (%)	530 (81.7)	237 (74.1)	143 (85.1)	150 (93.2)
Mean (SD)	¥2044.4 (4872.3)<br>$12 994.3 (30 968.7)	¥1223.5 (2601.4)<br>$7776.6 (16 534.7)	¥2362.9 (4973.7)<br>$15 018.8 (31 613.2)	¥3037.9 (6915.7)<br>$19 309.1 (43 956.7)
[Q1, Q3]	¥[175.3, 1715.5]<br>$[1114.2, 10 903.8]	¥[131.3, 1123.0]<br>$[834.6, 7137.9]	¥[214.4, 2457.1]<br>$[1362.7, 15 617.5]	¥[278.0, 2143.6]<br>$[1767.0, 13 624.9]
Cost of prescription drugs[a] (¥1000 PPPM)				
n (%)	649 (100.0)	320 (100.0)	168 (100)	161 (100.0)
Mean (SD)	¥839.3 (3754.8)<br>$5334.6 (23 865.8)	¥319.9 (1206.3)<br>$2033.3 (7667.3)	¥832.5 (3399.3)<br>$5291.4 (21 606.2)	¥1878.5 (6364.9)<br>$11 939.9 (40 455.7)
[Q1, Q3]	¥[26.1, 305.1]<br>$[165.9, 1939.2]	¥[13.5, 97.0]<br>$[85.8, 616.5]	¥[36.9, 479.3]<br>$[234.5, 3046.5]	¥[88.1, 760.6]<br>$[560.0, 4834.4]
Cost of stem cell transplants (¥1000 PPPM)				
n (%)	44 (6.8)	3 (0.9)	15 (8.9)	26 (16.1)
Mean (SD)	¥36.8 (69.2)<br>$233.9 (439.8)	¥151.5 (255.7)<br>$962.9 (1625.2)	¥32.9 (31.3)<br>$209.1 (198.9)	¥25.8 (27.4)<br>$164.0 (174.2)
[Q1, Q3]	¥[7.9, 41.5]<br>$[50.2, 263.8]	¥[0.06, 446.7]<br>$[0.4, 2839.3]	¥[7.8, 46.5]<br>$[49.6, 295.6]	¥[10.1, 29.3]<br>$[64.2, 186.2]
Cost of drugs[b] (¥1000 PPPM)				
n (%)	649 (100.0)	320 (100.0)	168 (100)	161 (100.0)
Mean (SD)	¥2914.1 (5594.8)<br>$18 522.2 (35 560.9)	¥1797.7 (3003.7)<br>$11 426.3 (19 091.7)	¥3488.5 (5385.1)<br>$22 173.1 (34 228.1)	¥4533.6 (8546.1)<br>$28 815.9 (54 319.6)
[Q1, Q3]	¥[489.4, 2826.9]<br>$[3110.7, 17 968.0]	¥[313.1, 1965.9]<br>$[1990.1, 12 495.4]	¥[801.8, 4206.4]<br>$[5096.3, 26 736.2]	¥[876.6, 3480.5]<br>$[5571.7, 22 122.3]
Cost of labs (¥1000 PPPM)				
n (%)	647 (99.7)	318 (99.4)	168 (100)	161 (100.0)
Mean (SD)	¥49.2 (55.9)<br>$312.7 (355.3)	¥47.0 (64.8)<br>$298.7 (411.9)	¥55.2 (54.7)<br>$350.9 (347.7)	¥47.3 (33.2)<br>$300.6 (211.0)
[Q1, Q3]	¥[16.5, 63.4]<br>$[104.9, 403.0]	¥[12.3, 59.0]<br>$[78.2, 375.0]	¥[19.1, 73.1]<br>$[121.4, 464.6]	¥[24.0, 59.5]<br>$[152.5, 378.2]
Cost of total[c] healthcare PPPM (¥1000 PPPM)				
n (%)	649 (100.0)	320 (100.0)	168 (100)	161 (100.0)
Mean (SD)	¥3341.4 (5654.6)<br>$21 238.2 (35 941.0)	¥2214.5 (3167.1)<br>$14 075.5 (20130.3)	¥3959.5 (5443.1)<br>$25 166.8 (34596.7)	¥4936.0 (8548.4)<br>$31 373.5 (54334.2)
[Q1, Q3]	¥[834.5, 3337.7]<br>$[5304.1, 21 214.6]	¥[512.8, 2667.2]<br>$[3259.4, 16 952.9]	¥[1175.1, 4726.5]<br>$[7469.0, 30 042.0]	¥[1197.9, 4244.4]<br>$[7613.9, 26 977.7]

Table S1. Overall All-Cause Treatment Costs in Patients with Peripheral T-cell Lymphoma

Treatment Costs	Overall Treated (N = 649)	1LOT (N = 320)	2LOT (N = 168)	≥3LOT (N = 161)
Cumulative[d] cost of PTCL-related healthcare per patient (¥1000)				
n (%)	649 (100.0)	320 (100.0)	168 (100)	161 (100.0)
Mean (SD)	¥62 323.9 (168 352.9)<br>$396 149.0 (1 070 209.6)	¥28 267.1 (82 696.3)<br>$179 685.1 (525 594.3)	¥65 456.2 (154 437.4)<br>$416 064.5 (981 650.9)	¥126 736.6 (264 159.6)<br>$805 606.5 (1 679 065.0)
[Q1, Q3]	¥[6434.4, 44 950.2]<br>$[40 891.8, 285 671.2]	¥[3892.4, 19 476.8]<br>$[24 739.0,123 789.6]	¥[10 208.7, 53 972.6]<br>$[64 881.0, 342 967.1]	¥[21 326.7, 96 124.4]<br>$[135 547.6, 610 993.0]

Abbreviations: ER, emergency room; LOT, line of therapy; PPPM, per patient per month; Q, quartile; SD, standard deviation.

[a] Prescription drugs include drugs for PTCL-related anticancer treatments (excluding steroids).

[b] Drugs include all drugs including anticancer drugs.

[c] Total healthcare includes all healthcare utilization including hospitalizations, ER visits, outpatient visits, prescription drugs, stem cell transplants, prescription and non-prescription drugs, and labs.

[d] From diagnosis to end of follow-up.
